# Begging behavior as an honest signal of need and parent–offspring association during the postfledging dependency period

**DOI:** 10.1002/ece3.5279

**Published:** 2019-06-17

**Authors:** Kayla L. Davis, Sarah M. Karpanty, Jeffrey A. Spendelow, Jonathan B. Cohen, Melissa A. Althouse, Katharine C. Parsons, Cristin F. Luttazi

**Affiliations:** ^1^ Department of Fish and Wildlife Conservation Virginia Tech Blacksburg Virginia; ^2^ Patuxent Wildlife Research Center U. S. Geological Survey Laurel Maryland; ^3^ Department of Environmental and Forest Biology State University of New York College of Environmental Science and Forestry Syracuse New York; ^4^ Massachusetts Coastal Waterbird Program Mass Audubon Cummaquid Massachusetts

**Keywords:** begging, honest signaling, parent–offspring conflict, postfledging parental care, roseate tern, *Sterna dougallii*

## Abstract

Honest signaling mechanisms can function to appropriate care to hungry offspring and avoid misdirected care of unrelated offspring. Begging, the behavior by which offspring solicit food and parental care, may be an honest signaling mechanism for need, as well as association of parents and offspring. Roseate terns (*Sterna dougallii*) exhibit prolonged parental care during the postbreeding staging period, offering an ideal system in which to study begging as an honest signaling mechanism. We conducted focal sampling during two premigratory staging seasons (2014 and 2015) at Cape Cod National Seashore, Massachusetts, USA to determine whether postfledging tern begging behavior was an honest signal for need and parent–offspring association. Based on honest signaling theory, we expected begging behavior to be highest during times of high perceived need, and we expected to see a decrease in begging behavior as young terns became increasingly independent of the care‐giving parent. Also, we predicted that young terns would be more likely to beg at parents than nonparents. We found that young roseate terns begged at their parents more often than nonparents; however, they did not always beg at parents. Model predictions of begging probability showed a linear relationship between begging and time of day and date of season, such that begging increased with time of day and decreased with date of season, respectively. Our results provide evidence for honest parent–offspring interactions and are inconsistent with parent–offspring conflict theory but suggest that begging may play a complex role in postfledging parent–offspring interactions.

**OPEN RESEARCH BADGES:**

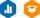

This article has been awarded Open Data, Open materials Badges. All materials and data are publicly accessible via the Open Science Framework at https://doi.org/10.5281/zenodo.2656718.

## 1 INTRODUCTION

Begging, the behavior by which offspring solicit food and parental care, is a good model for studying parent–offspring conflict (POC) and the evolution of signaling. According to POC theory, conflict arises over the amount of parental investment provided, with offspring soliciting more resources than parents are selected to give (Trivers, 1974). However, for this strategy to be evolutionarily stable, theory predicts that this behavior should be costly (Godfray, 1991). Costs of begging, which include energy expenditure (Godfray, 1991, 1995; Ogawa et al., 2015), loss of inclusive fitness (Rodríguez‐Gironés, Cotton, & Kacelnik, 1996), and predator and possibly parasite attraction (Tomás & Soler, 2016), may limit this behavior and increase its reliability as an honest parent–offspring signaling mechanism (Levréro, Durand, Vignal, Blanc, & Mathevon, 2009). Alternatively, an evolutionarily stable strategy may still be achieved in the absence of high costs if there is little to be gained from displaying the behavior dishonestly (Royle, Hartley, & Parker, 2002). For example, begging may not be a particularly costly behavior for fledged chicks, but this behavior may remain honest due to a relatively low payoff of cheating (e.g., begging at unrelated adults or soliciting more care than needed may not result in feeding; Zollman, Bergstrom, & Huttegger, 2013, Rich & Zollman, 2016).

In the context of parent–offspring communication and nestling need, begging behavior has been most studied as a behavior that is directed at parents and as a response to parental stimuli (Kilner & Johnstone, 1997). However, for fledglings of noncolonial species and for both nestling and fledglings in colonial species, there is opportunity to deceitfully beg for care from a nonparent (Jacot, Reers, & Forstmeier, 2010). Thus, begging could be used deceitfully, both by offspring in the context of POC, and by nonoffspring to obtain care from nonparents. In the face of these conflicting interests, reliable signaling mechanisms that enable effective parent–offspring communication are needed for adaptive parental responses (Godfray, 1995). Foremost among these is direction of parental care at appropriate young. In colonial species in particular, the need for parent–offspring recognition is high given the potential costs of misdirected feeding (Aubin & Jouventin, 2002; Levréro et al., 2009). Indeed, in some colonial species, there is evidence for embryonic vocal recognition of parents and offspring (Saino & Fasola, 2010), which likely functions, at least partially, to avoid misdirected parental care. Potential for deceit depends on the costs and benefits to parents of responding to and ignoring true and deceitful signals. For example, if adults rarely feed unrelated juveniles due to effective offspring recognition mechanisms, the juveniles have little to gain from begging at them. Given the evidence for parent–offspring recognition and the potential costs of offspring cheating (or lack of benefits from doing so), we expect that colonial breeding species with prolonged parental care should be capable of parent–offspring recognition and that begging behavior should be limited to one's own parents as an honest parent–offspring interaction.

There is a long history of avian research on begging in the context of POC; however, much of the literature focuses on this behavior in nestlings (but see Kouba, Bartos, & St'astny, 2014; Middleton, Green, & Krebs, 2007; Riou, Chastel, & Hamer, 2012) despite the occurrence of prolonged parental care in many species. In species such as seabirds, raptors, and others that exhibit specialized foraging techniques, parental care is prolonged during the postfledging period for many months (Ashmole & Tovar, 1968; Burger, 1980; Feare, 1975; Hewett Ragheb & Walters, 2011; Hunt, Holzhaider, & Gray, 2012; Limmer & Becker, 2009; Watson, Spendelow, & Hatch, 2012). During this prolonged period of parental dependency, hatch‐year (HY) birds learn specialized foraging techniques (Watson & Hatch, 1999) and other skills needed for functional independence, such as development of flight techniques essential for long dispersal or migratory movements. Parent–offspring interactions, including begging behaviors, are expected to change during the postfledging dependency period due to changes in the costs and benefits of parental care to both parents and offspring (Trivers, 1974). As fledglings approach functional independence, conflict arises over the duration of parental care provided (Trivers, 1974). As noted above, potential for deceit in signaling depends on the costs and benefits to parents of responding to and ignoring true and deceitful signals. For example, offspring may continue to solicit care from parents after reaching functional independence despite decreased need because parental delivery of food is more energetically efficient than self‐foraging (Middleton et al., 2007). However, if costs for ignoring offspring begging are low, parents may become increasingly aggressive toward offspring or less responsive to cues to limit parental investment during the postfledging dependency period (Davies, 1978), which could function to limit deceitful begging. If begging is an honest signal for need, we would expect this behavior to decrease over time as offspring become more capable of feeding themselves. However, if offspring behave consistently with POC theory, they may attempt to extend parental care past the point which is evolutionarily stable for parents to maximize their own fitness levels. Thus, we expect to see begging behavior continued throughout the postfledging dependency period followed by an increase in begging behavior as offspring near functional independence.

As noted above, we expect begging behavior to change with offspring ontogeny, but it is also possible that diurnal variation may exist in begging behavior related to offspring need. For species that forage by sight, it is unlikely that much if any feeding occurs overnight (Shealer & Kress, 1994). Thus, if begging is an honest signal of need, offspring may increase frequency of begging behavior before nightfall if they are not fed at night because their perceived need for food is highest before a night‐time fast (Montevecchi & Porter, 1980). However, if offspring behave deceitfully, as would be expected under POC theory, we might see no diurnal directionality in begging behavior but instead see a uniform distribution of begging behavior over the course of a day, such that begging is equally likely at all times of the day.

The federally endangered Northwest Atlantic population of roseate terns (*Sterna dougallii dougallii*) provides an ideal model for studying honesty of begging signals by HY birds because they exhibit prolonged parental care, postfledging care has a high benefit to offspring, and parent–offspring interactions are easily observed during most of their long staging season (Shealer & Burger, 1995; Watson et al., 2012). This species breeds colonially on coastal islands from Nova Scotia to New York and Connecticut. During the postbreeding dispersal period, roseate tern adults and HY birds depart the breeding grounds in paired groups; the adult male departs with the A‐chick (i.e., first that hatched in a brood) and attends it during staging, and the female departs with and attends to the B‐chick if it survives to fledging (Watson et al., 2012). The adult‐HY pairs fly to beaches and islands around Cape Cod, MA, where they stage before departing on fall migration to South America (Watson et al., 2012). Roseate terns do not regurgitate fish meals to their offspring like some other seabirds; instead, HY begging serves to initiate fishing by the attending adult. Upon return to the staging area with a fish, the adult calls the HY tern away from the staging flock to feed it (Shealer & Kress, 1994). Roseate tern staging duration can last at least 1.5 months (between mid‐July and late‐September; Davis, 2016). HY roseate terns are dependent on the male or female care‐giving adults for much of that time (Shealer & Burger, 1995; Watson & Hatch, 1999) until they join parents on fishing trips in offshore areas late in the staging season. Additionally, HY terns continue to grow throughout the staging season (LeCroy & Collins, 1972; Schauroth & Becker, 2008; Stienen & Brenninkmeijer, 2002). For many tern species, including roseate terns, parental care is prolonged through staging and often into migration and wintering, presumably to allow time for HY terns to learn specialized foraging skills (Ashmole & Tovar, 1968; Burger, 1980; Feare, 1975; Shealer & Burger, 1995). HY roseate tern begging behavior can be observed at the Cape Cod staging grounds well into the staging period. It is worth noting that feeding is less frequently observed because HY roseate terns often are called away from the flock by the parent before they are fed and roseate terns fish in offshore areas away from staging flocks; thus, most observable interactions at staging sites between offspring and parents are begging events.

**Figure 1:**
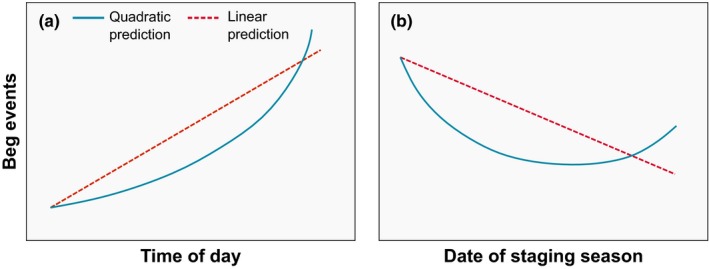
(a) Predicted distribution for begging events by hatch‐year roseate terns during fall premigratory staging by time of day. We expected begging events to be most prevalent in the evenings when offspring needs are highest. We tested for a quadratic relationship between begging and time of day, such that we would expect to see an increase of the rate of begging with time of day. We also tested for a linear relationship between begging and time of day, where we expected to see a constant increase in begging behavior with time of day. (b) Predicted distribution for begging events by date of staging season. We also tested for quadratic and linear relationships between begging behavior and date of season. If our hypothesis about parent–offspring conflict is supported, we expect to see a decrease in begging events over time as offspring needs decrease, but we expect to see a period of elevated begging at the end of the staging season as offspring begin foraging for themselves and attempt to prolong parental care before they become functionally independent (quadratic prediction). The linear relationship prediction represents our expectation if begging behavior is an honest signal of need and the end of the staging season is not a period of elevated parent–offspring conflict

We studied HY roseate tern begging behavior during two postbreeding staging seasons (2014 and 2015) at Cape Cod National Seashore (CCNS). Our objectives were to (a) examine whether HY roseate terns begged at their attending parent more often than nonparents, and (b) determine how temporal variables (date of staging season and time of day) affected begging behavior. Given the evidence for parent–offspring vocal recognition and potential costs and benefits associated with honest and deceitful communication by offspring, we hypothesized that: (a) HY roseate terns would beg at their own parent more frequently than at nonparents; (b) begging is an honest signal of need and therefore would be most frequently observed in the evening (when HY tern perceived needs were highest before night‐time fasting; Figure 1a). We predicted that this relationship could follow a linear trend, such that begging behavior increases at a constant rate throughout the day (Figure 1a: linear prediction), or it could follow a quadratic trend, such that the rate of begging increases more quickly at the end of the day relative to earlier times (Figure 1a: quadratic prediction). We also hypothesized that (c) HY roseate terns would beg at their parent less frequently as they reached functional independence, such that begging would decrease over the staging season (Figure 1b: linear prediction); and (d) based on POC theory, we predicted a late, brief period of deceit where begging increases as HYs attempt to extend parental care when they start fishing for themselves (Figure 1b: quadratic prediction). Alternatively, we also would expect to see a similar pattern in begging behavior if the end of the staging period represents a period of increased HY need as a result of inefficient self‐foraging. However, as noted previously, there is evidence from other species with specialized foraging techniques that parental care and feeding of offspring can be prolonged for 6 months or more postfledgling (Ashmole & Tovar, 1968; Burger, 1980; Feare, 1975; Hewett Ragheb & Walters, 2011; Hunt et al., 2012; Limmer & Becker, 2009; Watson et al., 2012). Although we are unable to definitively disentangle need from deceit under hypothesis four, we think it is unlikely, given the evidence for prolonged parental feeding in terns and other species, that an increase in begging behavior at the end of the staging season would be strong evidence for an increase in HY need. Nevertheless, we conservatively suggest that support for hypothesis four could indicate need or deceit in this context. As an approximation to these predictions for hypothesis four, we expected to see a concave curvature in the relationship between begging behavior and date of staging season, such that begging behavior is relatively high at the beginning of the season, decreases with date, and increases toward the end of the staging season (Figure 1b: quadratic prediction). If our predictions for hypotheses 1–3 are upheld, we would conclude that begging in HY roseate terns is an honest signaling mechanism. Support for hypothesis four could indicate honest or deceitful behavior. We test these hypotheses in a generalized mixed modeling framework where we model the linear and quadratic effects of time of day and date of staging season to evaluate the relationship between begging behavior and time of day/date of staging season.

## 2 METHODS

### 2.1 Study area

**Figure 2:**
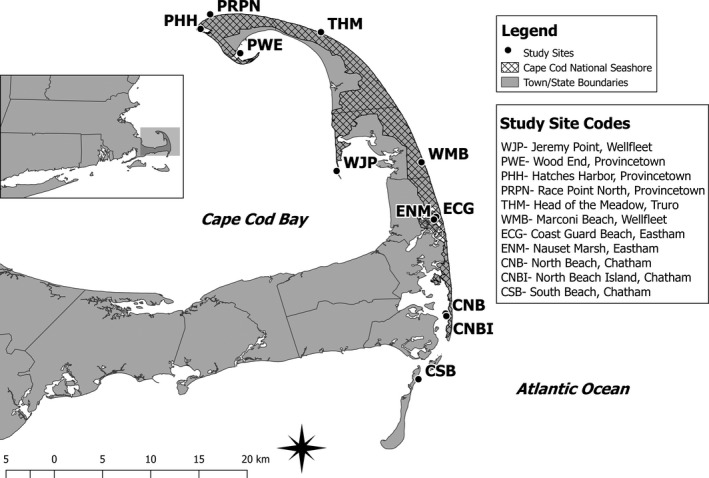
Cape Cod National Seashore boundaries and the survey sites

Cape Cod National Seashore encompasses over 180 km^2^ of marine, estuarine, fresh water, and terrestrial ecosystems (National Park Service, 2014). Each year in late summer and early fall (mid‐July–late‐September), thousands of terns, including the federally endangered NW Atlantic roseate terns and common terns (*S. hirundo*), as well as many other migrant shorebirds, convene at CCNS to stage for their postbreeding migration. Staging areas at CCNS consisted of tidal flats, sandbars and sand spits, and barrier island beaches. We selected sites within CCNS based on preliminary field observations indicating significant and consistent roseate tern use within and across seasons (Figure 2; Jedrey, Harris, & Ray, 2010).

### 2.2 Field methods

**Figure 3:**
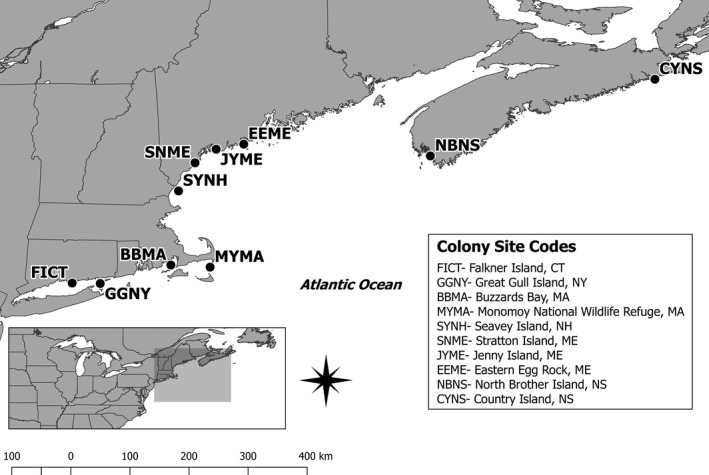
Northwest Atlantic roseate tern colony sites active (one or more roseate tern nests present) during the 2014 and 2015 breeding seasons. Managers at all colony sites except those in Buzzards Bay, MA (BBMA) banded hatch‐year terns with coded plastic field‐readable bands

All HY terns in our study were banded during 2014 and 2015 breeding seasons preceding our study period by colony managers and staff at nine of the 12 then active NW Atlantic roseate tern colonies (Figure 3). Roseate tern chicks were banded with uniquely coded plastic field‐readable leg bands. The banding protocol varied between colonies by chick capture method and band application method (i.e., leg banded, type of glue used to close bands, etc.), but variations in methods among colonies were minor. In 2014 and 2015, adult roseate terns also were banded with uniquely coded plastic field‐readable leg bands, and prior to 2014, adults were banded with unique combinations of plastic color bands and uniquely coded metal bands (Spendelow et al., 2016).

At the CCNS staging grounds, we located mixed‐species tern flocks at the sites identified by previous and concurrent work. Our main goal was to locate large mixed‐species flocks containing high proportions of roseate terns because these offered the greatest opportunity for observing large numbers of roseate terns and many HY‐adult interactions. A two‐observer team visited 2‒4 sites per day to search for staging flocks. The number of sites visited each day was dependent on the size of flocks at each site, timing of the tidal cycle at each site, weather, and day length. After locating staging flocks, we approached flocks on foot and identified banded individuals with spotting scopes (Althouse et al., 2016). Usually, observers were within approximately 40 m of flocks to conduct band resighting and behavioral observations (Althouse et al., 2016). We conducted 5‐min focal samples (Altmann, 1974) of banded HY roseate terns to investigate the relationship between date of season (day of year beginning on 23 July [204] in both seasons through 23 September [265] in 2014 and 22 September [264] in 2015) and time of day (24‐hr scale) with HY tern begging behavior. We use date of season as a proxy for HY age because age was unknown, but we expected age and date of season to be highly correlated. Most HY terns were banded soon after hatch; however, age at banding was not recorded in most cases. Days since banding was our closest approximation of HY roseate tern age; thus, we used this metric to run a Pearson correlation test for date of season and days since banding for HY roseate terns with banding date records. See Appendix 1 for methods and results of this correlation test.

The number of focal samples recorded at each site varied depending on the number of banded HY roseate terns in the flock. During focal samples, observers used spotting scopes to identify and watch banded focal HY, continuously recording begging and feeding behaviors. If HY begging or feeding behaviors were directed at another individual, we recorded the identity of the associated bird (unmarked, color band combination, or field‐readable code). We used these interactions to determine whether HY roseate terns preferentially begged at parents or whether begging behavior was directed at presumably unrelated adults. We also recorded aggressive interactions between adults and HY terns, including pecking and chasing directed at HY terns. We terminated a focal sampling session after all HY roseate terns had been focal sampled once, or resighting conditions became unfavorable due to poor lighting, weather, or an incoming tide.

### 2.3 Analytical methods

To address our first hypothesis about the probability that HY begging is directed at parents versus nonparents, we created a subset of our dataset of focal HY roseate terns that included only birds with known, uniquely marked parents that had been observed begging at an adult at least once. We obtained parent identities from tern colony managers who confirmed parenthood by trapping at the nest or observation of banded birds at the nest. Using the subset of HY roseate terns with an identified parent, we determined if begging behaviors were directed at parents or nonparents. We fit an intercept‐only logistic regression, where begging at a parent (1) versus a nonparent (0) was the binary dependent variable, to determine whether the probability of HY roseate tern begging at parents was greater than the probability of begging at nonparents. In this model, the estimate for the intercept indicates the probability of success (begging directed at true parent), and the *p*‐value indicates whether this probability estimate is significantly different than zero (or 50% after logit transformation). Thus, we evaluate whether HY terns beg at parents more than nonparents and whether this probability is significantly different than 50%. The random effect of individual was included to account for repeated observations of the same HY individual. There were no begging interaction observations of the same adult birds in the subset of our dataset between years. This analysis was conducted in Program R v3.3.3 (R Core Team, 2015) using package lme4 (Bates, Maechler, Bolker, & Walker, 2015).

We addressed our remaining hypotheses about the effects of temporal variables (time of day and date of staging season) on begging behavior with our full dataset, and we totaled the number of begging events during each 5‐min focal sample for all individuals observed. A Poisson distribution is commonly used to model count data; however, this distribution can be restrictive for ecological applications because it assumes that the mean and variance are equal (Zuur, Ieno, Walker, Savaliev, & Smith, 2009). Ecological count data are often overdispersed, such that the variance is greater than the mean (Zuur et al., 2009), as was the case with our observations of begging events. The negative binomial distribution is a good alternative for overdispersed data or data with many zeros (Zuur et al., 2009). Therefore, we used mixed‐effects negative binomial regression in Program R v3.3.3 (R Core Team, 2015) and package glmmADMB (Fournier et al., 2012) using the maximum likelihood with a trust region algorithm to model the effects of time of day and date of staging season (and the associated quadratic effects) on the number of HY roseate tern begging events during 5‐min focal samples. We included the random effects of individual and survey site to control for nonindependence of behaviors for each HY tern and possible nonindependence in begging behavior between sites, respectively. We also evaluated a null model that included an intercept and our random effect terms. We combined data from our two seasons of data collection into a single analysis, as there were no focal animals sampled in both years. All covariates modeled as fixed effects were standardized by subtracting the mean and dividing by the standard deviation for the entire dataset so relative effect sizes could be intuitively interpreted across all predictor variables. We conducted model selection with our three candidate models that represented our hypotheses regarding the relationship between begging behavior and time of day/date of staging season to determine which hypothesis was best supported by the data. We ranked our models using AIC corrected for small sample bias (AIC_c_: Burnham & Anderson, 2002). We present model predictions from our top‐ranked model, and we used the ggplot2 package (Wickham, 2009) to plot our results. All means are presented as ±1 SE unless otherwise noted.

## 3 RESULTS

We were able to determine the identities of one or both parents for 119 HY roseate terns out of 2,328 HY roseate terns banded in 2014–2015, and we used this subset of our full dataset in our logistic regression analysis to assess whether HY terns preferentially begged at parents or nonparents. We observed 15 of these 119 HY terns begging at adults on the CCNS staging grounds, and we recorded 34 begging events from these 15 HY terns. Our logistic regression analysis showed that 86% (95% confidence interval: 85.5%–86.4%) of HY roseate tern beg interactions (from our reduced dataset of HY terns with known parents) were directed at parents over nonparents (Intercept = 1.8 ± 0.7; *p* = 0.01), and there was little additional variability in the model due to begging behavior by individual HY tern (σ = 0.2 ± 0.06).

**Figure 4:**
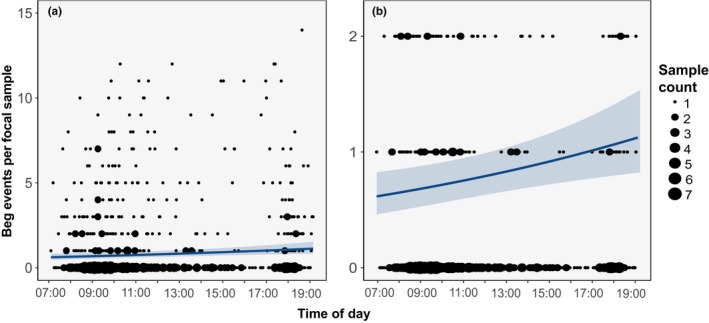
Hatch‐year roseate tern begging behavior by time of day (24‐hr scale) at Cape Cod National Seashore, MA during the 2014–2015 postbreeding, premigratory staging seasons. Begging behavior increased linearly with time of day, as illustrated by negative binomial model predictions (blue line) and 95% confidence intervals (blue ribbon) for the effect of time on hatch‐year tern begging, in which begging behavior was modelled as the count of beg events observed during a 5‐min focal sample. Black circles are focal sample data indicating the number of beg events per focal sample at each sample time. Relative size of the circle indicates the number of focal samples. The full dataset used for modeling the relationship between time of day and begging behavior is shown in (a), and (b) is reduced to better show the linear relationship

**Figure 5:**
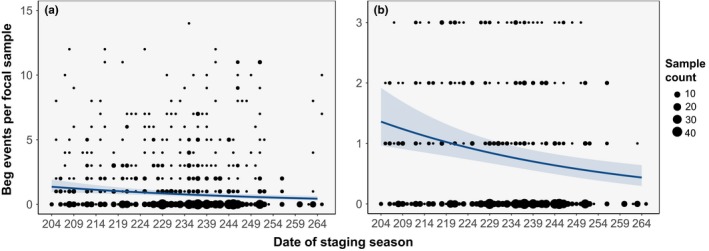
Hatch‐year roseate tern begging behavior by date of staging season at Cape Cod National Seashore, MA during the 2014–2015 postbreeding, premigratory staging seasons. Begging behavior decreased linearly with date of staging season, as illustrated by negative binomial model predictions (blue line) and 95% confidence intervals (blue ribbon) for the effect of date on tern begging, in which begging was modelled as the count of beg events observed during a 5‐min focal sample. Predicted begging events by date of the stopover season decreases from day 204 (July 23) to day 265 (2014: September 23; 2015: September 22 [59 sampling days]), such that the frequency of begging decreases with date. Black circles are focal sample data indicating the number of beg events per focal sample at each sample date. Relative size of the circle indicates the number of focal samples. The full dataset used for modeling the relationship between date of season and begging behavior is shown in (a), and (b) is reduced to better show the linear relationship

Our full dataset consisted of 970 5‐min focal samples from 664 HY roseate terns during the 2014–2015 staging seasons. Focal sample observation times ranged from 07:11 to 19:22 EST. Mean focal sample time was 11:59 EST. Focal sample observations were recorded on 61 days in 2014 (23 July–23 September) and 60 days in 2015 (23 July–22 September). Mean focal sample date was 22 August for both years. Aggressive interactions between adults and HY terns were observed only 16 times and were not included in any analyses due to lack of data. Mean beg events observed in a focal sample was 1.12 ± 0.16. We used this full dataset for the remainder of our analyses to determine the effects of time of day and date of staging season on HY tern begging behavior. Our top model included the linear‐effect terms for time of day and date of staging season (Table 1), and we used this model to make predictions. The fixed‐effect terms of time of day and date of staging season accounted for 11% of the variation in the data, and the full model, including the random‐effect terms of individual HY tern and staging site accounted for 96% of the variation in the data. Model estimates indicated that begging behavior increased by 20% with every one standard deviation increase in time of day (*p* = 0.002; Table 2), and begging events per focal sample decreased by 21% with every one standard deviation increase in date of staging season (*p* < 0.001; Table 2). Model predictions for the fixed‐effect terms show the linear relationship between begging events per focal sample and time of day (Figure 4) and date of staging season (Figure 5).

**Table 1:** Model structure, information‐theoretic model selection criteria, and parameter counts for negative binomial mixed models of hatch‐year roseate tern begging behavior at Cape Cod National Seashore, 2014–2015

Model	ΔAIC_c_ a	*w_i_* b	Relative likelihood	Deviance	*K* c
Date^d^ + time^e^	0.00	0.74	1.00	2445.58	6
Date + time + date^2^ + time^2^	2.04	0.26	0.36	2443.56	8
Null model	19.25	0.00	0.04	2468.88	4

Notes

We modeled begging as the number of begging events recorded during a 5‐min focal sample. All models included random effects of individual bird and site.

a Akaike's Information Criterion corrected for small sample bias. Top model AIC_c_ was 2457.67.

b Model weight.

c Number of parameters included in the model.

d Date of the staging season (numbered 204–265, 2014; 204–264, 2015).

e Time of day (modelled as time on a 24‐hr scale: 07:11‒19:22).

**Table 2:** Negative binomial mixed model estimates of hatch‐year roseate tern begging behavior at Cape Cod National Seashore, 2014–2015

Model term	Estimate	*SE* a	Lower 95% CI^b^	Upper 95% CI
Intercept	‒0.25	0.11	‒0.48	‒0.02
Date^c^	‒0.24	0.06	‒0.37	‒0.12
Time^d^	0.17	0.06	0.06	0.29

Note

We report coefficient values for our top‐ranked model from information‐theoretic model selection (*w_i_* = 0.74). We modeled begging as the number of begging events recorded during a 5‐min focal sample. All models included random effects of individual HY roseate tern and sample site.

a Standard Error.

b Confidence Interval.

c Date of the staging season (numbered 204–265, 2014; 204–264, 2015).

d Time of day (modeled as hour on a 24‐hr scale: 07:11‒19:22.

## 4 DISCUSSION

Our study objectives were to (a) examine whether HY roseate terns begged at their attending parent more often than nonparents, and (b) determine how temporal variables (date of staging season and time of day) affected begging behavior. Under the honest signaling hypothesis, we predicted that HY roseate terns should beg at parents more than nonparents and that HY tern begging should be consistent with their perceived need, such that begging should increase with time of day and decrease with date of season. We found that probability of begging at a true parent was higher than begging at a nonparent and that HY roseate tern begging behavior increased linearly with time of day and decreased linearly with date of staging season.

Our results are consistent with our hypothesis that roseate terns, a colonially breeding species with prolonged parental care, should be capable of parent–offspring recognition and that HY begging behavior should be limited to their own parents (Aubin & Jouventin, 2002; Levréro et al., 2009; Saino & Fasola, 2010). Parental recognition of offspring has been studied in many taxa and in several different contexts including timing of recognition development (Saino & Fasola, 2010), functionality of parental recognition of offspring (Godfray, 1991; Goncharova, Klenova, & Bragina, 2015; Lefevre, Montgomerie, & Gaston, 1998; Middleton et al., 2007; Ringler, Pašukonis, Ringler, & Huber, 2016), and mechanisms by which recognition occurs (Aubin & Jouventin, 2002; Goncharova et al., 2015; Levréro et al., 2009; Padilla de la Torre, Briefer, Ochocki, McElligott, & Reader, 2016). Our results support existing theory for parent–offspring recognition/discrimination that is well established in many taxa. Indeed, in colonially breeding species, like roseate terns, parents should discriminate their mobile offspring from others' during both pre‐ and postfledging periods to avoid costs of misdirected parental care (Aubin & Jouventin, 2002; Levréro et al., 2009). In such a situation, offspring cheating may be rare because it is unlikely that a nonparent will be fooled by a dishonest HY; thus, there may be few benefits to gain from cheating (Rich & Zollman, 2016; Zollman et al., 2013). Although we found little evidence of adult aggression toward offspring in our system, it is possible that aggressive interactions between nonparents and cheating offspring may mediate the honesty of begging at parents versus nonparents (Davies, 1978); however, we were unable to evaluate this hypothesis with our data. Here, we demonstrate that HY roseate tern begging is more frequently directed at the HY's own parents versus unrelated adults; therefore, we conclude that the high cost of misdirected parental care and the relative lack of benefits to gain from cheating has resulted in honest communication, that is, parent–offspring recognition.

Under the honest signaling hypothesis, we predicted that HY roseate tern begging would be highest in the evening, before night‐time fasting, if it were an honest signal of need. We modeled a linear and quadratic relationship between begging behavior and time of day to determine the shape of this relationship and whether begging increased gradually throughout the day or accelerated more quickly at the end of the day, respectively. We found that HY tern begging increased linearly with time of day, such that the frequency of begging increased consistently throughout the day and peaked at the end of the day. It is unlikely that much if any feeding occurs overnight because roseate terns fish by sight (but see Niethammer & Patrick, 1998). Only on the most well‐lit full moon nights would it be feasible for roseate terns to successfully fish during the night hours. Therefore, HY roseate terns may beg most frequently at dusk because it typically is their last time to be fed before nightfall (Shealer & Kress, 1994) and is part of preroosting activity. In addition, it is also possible that HY terns are fed predawn or shortly after dawn (Stienen et al., 2000), corresponding with the vertical diel migration of sand lance (*Ammodytes spp*.; Friedlander et al., 2009). If this assumption is correct, the observed increase in begging behavior throughout the day may indicate increasing hunger levels throughout the day. We rarely observed feeding and our morning observations did not begin early enough for us to test this hypothesis; however, this would be further evidence to support the hypothesis that HY tern begging behavior is an honest signal of need. The observed trend in begging throughout the day is consistent with our prediction for begging behavior as an honest signal of need; thus, we conclude that the relationship between time of day and beg events is indicative of honest signaling.

During the postfledging period, POC is thought to mediate parent–offspring association and the timing of offspring independence (Muriel, Ferrer, Balbontín, Cabrera, & Calabuig, 2015; Smiseth, Darwell, & Moore, 2003; Trillmich, Spiller, Naguib, & Krause, 2016). In our study, we observed a linear decrease in HY roseate tern begging behavior as the staging season progressed, likely indicating increasing independence of HY terns and further supporting our finding that HY tern begging is an honest signal of need. This finding is inconsistent with our prediction of a quadratic relationship between begging behavior and date of staging season and provides evidence against an elevated period of POC during the roseate tern postfledging dependency period. Moreover, honest signaling is more likely under uniparental care of single offspring (Royle et al., 2002), which is the case for roseate terns during the premigratory period, and this relationship has low potential for POC. HY ROST begging behavior continues throughout the staging period, albeit at decreased frequency as the season progressed, which may indicate a “soft‐independence” period at the end of the staging season. During this time, HY terns may begin to fish for themselves but maintain a parental bond for limited care before they reach functional independence. Additionally, these results could also support evidence for a prolonged parent–offspring bond poststaging. This suggests that for roseate terns, begging behavior is indeed an honest signaling mechanism and the continued, yet lower begging behavior indicates increasing foraging proficiency of HY roseate terns and a soft independence at the end of the staging season as HY terns continue to solicit parental care before they become completely independent of care‐giving parents.

Although our study could not specifically investigate responsiveness of adults to HY begging interactions, which may partially mediate the frequency with which offspring beg at parents (Middleton et al., 2007), we found little evidence of parental aggression toward HY birds during the postfledging dependency period. Due to the difficulty of observing roseate tern feeding behavior, we were unable to investigate whether adults' refusal to feed HY birds or the rate of feeding throughout the staging season may play a role in mediating begging frequency. However, these data could elucidate whether timing of independence is under offspring or parental control and uncover the function of begging in late‐staging season parent–offspring interactions (Muriel et al., 2015; Smiseth et al., 2003; Trillmich et al., 2016).

Another possible explanation for the lower yet continued begging behavior at the end of the staging season may be to reinforce the parent‐HY bond prior to southward migration. Although it is unknown whether parents and HY birds migrate together or remain together in winter quarters, observations of color‐marked roseate terns from the Azores population have indicated that HY terns continue to be fed in winter quarters until at least seven months of age (Nisbet, Gochfeld, & Burger, 2014). Thus, it is possible that postfledging begging behavior has multiple functions, including indicating need as well as reinforcing parent‐HY bonds. Future work should focus on parental responses to HY tern signals throughout the staging period and HY tern fishing behavior, particularly parental feeding rates as the staging season progresses and timing of HY tern fishing trips and fishing success rates, respectively. These data could yield a better understanding of the role of begging in parent–offspring communication as HY roseate terns become increasingly independent of parents. These data also could provide evidence for evolutionary pathways of begging behavior in species using various provisioning styles and parent‐versus offspring‐mediated timing of offspring independence. Nonetheless, we determined that HY roseate terns beg at their parents more than at nonparents and that begging behavior is affected by temporal factors, including time of day and date of staging season. Therefore, costs associated with (or relative lack of benefits to be gained from) begging may limit this behavior, resulting in relatively honest interactions between parents and offspring in roseate terns. These results support our hypotheses about honesty of interactions between roseate tern parents and offspring; however, we provide evidence against POC during the postfledging dependency period. Our observations suggest instead that begging could have multiple functions, particularly if HY birds continue to depend on parents during migratory and wintering periods.

## CONFLICT OF INTEREST

All authors have no significant competing financial, professional, or personal interests that might have influenced the performance or presentation of the work described in this manuscript.

## AUTHOR CONTRIBUTIONS

KLD, SMK, JAS, JBC, and KCP conceived and planned the field study. KLD carried out field work, data analysis, and took the lead in writing the manuscript. SMK, JAS, JBC, MAA, and KCP provided theoretical and analytical feedback. CFL provided substantial assistance with data management. All authors provided critical feedback and helped shape the research, analysis, and manuscript.

## Figures and Tables

**Figure A1:**
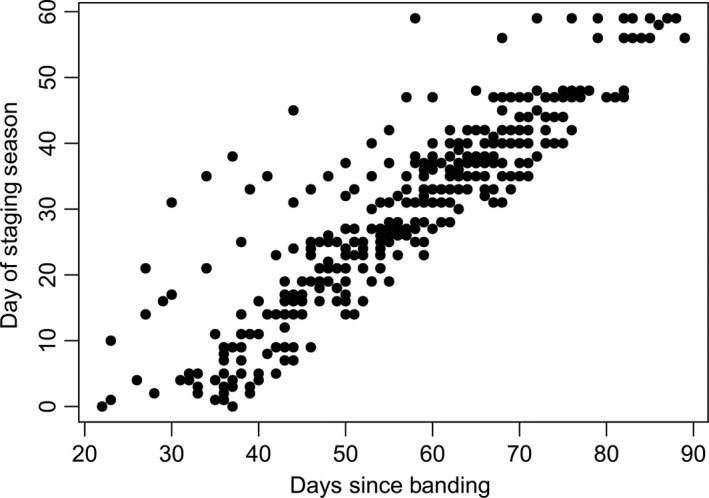
Correlation of date of staging season (day of year beginning on 23 July [204] in both seasons through 23 September [265] in 2014 and 22 September [264] in 2015) and days since banding for 504 hatch‐year roseate terns observed at the Cape Cod National Seashore staging grounds during the 2014 and 2015 seasons

## Data Availability

The authors have archived data and code used for analyses reported in this manuscript on GitHub (https://github.com/davisk93/ROST-Begging-Behavior) and registered a DOI for data and code on Zenodo (https://doi.org/10.5281/zenodo.2656718).

## APPENDIX 1

We used date of season as a proxy for HY age because age was unknown, but we expected age and date of season to be highly correlated. Most HY terns were banded soon after hatch; however, age at banding was not recorded in most cases. Days since banding was our closest approximation of HY roseate tern age; thus, we used this metric to run a Pearson correlation test between date of season and days since banding for HY roseate terns with banding date records.

Of the 970 HY roseate terns in our full dataset, 504 had available banding date records. We ran a Pearson correlation test on these 504 individuals to compare their observation date (date of staging season) and the days since they were banded. As expected, date of staging season and days since banding were highly positively correlated (*r*_502_ = 0.90, *p* ≤ 0.001; Figure A1). Therefore, we concluded that date of staging season was a reasonable approximation of age and allowed us to use our full dataset of 970 individuals for modeling the effects of date and time on HY roseate tern begging behavior.
